# Increased Intraepithelial Vα24 Invariant NKT Cells in the Celiac Duodenum

**DOI:** 10.3390/nu7115444

**Published:** 2015-10-30

**Authors:** Enrique Montalvillo, David Bernardo, Beatriz Martínez-Abad, Yessica Allegretti, Luis Fernández-Salazar, Carmen Calvo, Fernando G. Chirdo, José A. Garrote, Eduardo Arranz

**Affiliations:** 1 Mucosal Immunology Lab, IBGM, University of Valladolid-CSIC, Sanz y Forés 3, 47003 Valladolid, Spain; emontalvillo@gmail.com (E.M.); beamaraba@gmail.com (B.M.A.); jagarrote@saludcastillayleon.es (J.A.G.); 2 Antigen Presentation Research Group, Imperial College London, Northwick Park & St Mark’s Campus, Level 7W, St Mark's Building Watford Road Harrow HA1 3UJ, UK; d.bernardo.ordiz@gmail.com; 3 Gastroenterology Unit, Hospital Universitario de La Princesa and Instituto de Investigación Sanitaria Princesa (IIS-IP), Centro de Investigación Biomédica en Red de Enfermedades Hepáticas y Digestivas (CIBEREHD), Madrid 28006, Spain; 4 Laboratorio de Investigación en el Sistema Inmune –LISIN, Departamento de Ciencias Biológicas, Facultad de Ciencias Exactas, Universidad Nacional de La Plata, 115, La Plata 1900, Buenos Aires, Argentina; yallegretti@gmail.com (Y.A.); fchirdo@biol.unlp.edu.ar (F.G.C.); 5 Gastroenterology Unit, Hospital Clínico Universitario; Ramón y Cajal 3, Valladolid 47005, Spain; luisfernsal@gmail.com; 6 Paediatric Unit, Hospital Clínico Universitario; Ramón y Cajal 3, Valladolid 47005, Spain; carmencalvoromero@gmail.com; 7 Medical Laboratory Service, Hospital Universitario Rio Hortega; Dulzaina 2, Valladolid 47012, Spain

**Keywords:** Celiac Disease, Intraepithelial Lymphocytes, iNKT, Vα24-Jα18, IFNγ, Celiac Disease-like molecular profile

## Abstract

Celiac Disease (CD) is an interferon (IFN)γ-mediated duodenal hypersensitivity to wheat gluten occurring in genetically predisposed individuals. Gluten-free diet (GFD) leads to a complete remission of the disease. Vα24-restricted invariant NKT (iNKT) cells are important to maintain immune homeostasis in the gut mucosa because of their unique capacity to rapidly produce large quantities of both T-helper (Th)1 and Th2 cytokines upon stimulation. We studied the presence of these cells in the CD duodenum. Duodenal biopsies were obtained from 45 untreated-CD patients (uCD), 15 Gluten Free Diet-CD patients (GFD-CD), 44 non-inflamed non-CD controls (C-controls) and 15 inflamed non-CD controls (I-controls). Two populations from Spain and Argentina were recruited. Messenger RNA (mRNA) expression of Vα24-Jα18 (*invariant* TCRα chain of human iNKT cells), IFNγ and intracellular transcription factor Forkhead Box P3 (Foxp3), and flow cytometry intraepithelial lymphocyte (IEL) profile were determined. Both uCD and GFD-CD patients had higher Vα24-Jα18 mRNA levels than non-CD controls (I and C-controls). The expression of Vα24-Jα18 correlated with Marsh score for the severity of mucosal lesion and also with increased mRNA IFNγ levels. uCD and GFD-CD patients had decreased mRNA expression of FoxP3 but increased expression of Vα24-Jα18, which revealed a CD-like molecular profile. Increased numbers of iNKT cells were confirmed by flow cytometry within the intraepithelial lymphocyte compartment of uCD and GFD-CD patients and correlated with Vα24-Jα18 mRNA expression. In conclusion, we have found an increased number of iNKT cells in the duodenum from both uCD and GFD-CD patients, irrespective of the mucosal status. A CD-like molecular profile, defined by an increased mRNA expression of Vα24-Jα18 together with a decreased expression of FoxP3, may represent a pro-inflammatory signature of the CD duodenum.

## 1. Introduction

Celiac disease (CD) is an inflammatory disorder of the small intestine induced by wheat gluten and other prolamins from rye, barley and some varieties of oats [1] in genetically susceptible individuals. It is characterized by an interferon (IFN)-γ mediated type I cytokine profile [2]. CD manifestation is characterized by an increased number of intraepithelial and lamina propria lymphocytes, villous atrophy, tissue remodeling and the presence of anti-transglutaminase antibodies [3]. At present, the only treatment for CD is a life-long strict gluten-free diet (GFD), which normally leads to a complete remission of the disease [4].

Gut intraepithelial lymphocytes (IEL) comprise a heterogeneous population of cells outside the normal circulation. In addition to conventional T lymphocytes (CD3^+^ TCRαβ^+^, either CD4^+^ or CD8^+^) with an outstanding CD3^+^ CD8^+^ prevalence, Natural Killer (NK) cells and unconventional IEL populations such as CD8αα, TCRγδ^+^ cells, CD3^+^CD4^−^CD8^−^ cells and NKT lymphocytes are widely represented [5,6].

Increased total numbers of IEL CD3^+^ (both classical and TCRγδ^+^) and decreased IEL non-T cells (CD3^−^, CD103^+^) have been consistently reported in CD [7,8,9]. A direct cytolytic effect of conventional CD3^+^αβ^+^CD8^+^ cytotoxic IEL on adjacent enterocytes is undisputed [10,11] and related to villous atrophy [7]. However, the role of IEL TCRγδ^+^ in CD pathogenesis remains elusive, although they may have a key role in oral tolerance as supported by the identification of a subset of regulatory TCRγδ^+^ IEL population capable of limiting the cytotoxicity of IEL in CD treated on a GFD [12].

Classical regulatory Tcells (Tregs; CD3^+^, CD4^+^, CD25^+^ and intracellular transcription factor Forkhead Box P3^+^ (FoxP3^+^)) and non-classical interleukin (IL)-10 producing regulatory cells (Tr1: CD3^+^, CD4^+^, CD25^−^ and intracellular FoxP3^−^) are the main regulatory T-cells found in the intestine [13]. Tregs elicit their function by suppressing IL-2 production and T-cell proliferation [14], while Tr1 cells are the main source of IL-10 in the intestinal lamina propria since they are chronically stimulated and limit the production of pro-inflammatory cytokine by controlling inflammatory responses to dietary antigens. Compared to Tregs, the finding of larger numbers of Tr1 in the intestinal lamina propria suggests that these cells have an important regulatory capacity [15,16].

Invariant NKT cells (iNKT; CD3^+^, TCR Vα24^+^Vβ11^+^) are also important to maintain immune homeostasis [13]. Human iNKT cells express classical NK cell markers as well as an *invariant* TCRα chain (iNKTα) (Vα24-Jα18 in humans) paired to “semi-invariant” TCRβ chains (iNKTβ), which recognizes antigens presented by the major histocompatibility complex (MHC) class I-like molecule CD1d [17,18]. For all iNKT-cell TCRs, binding to CD1d is primarily mediated by the Vα-Jα rearranged *invariant* CDR3α loop [19]. Therefore, the anti-Vα24-Jα18 is the standard method used to detect human iNKT cells [20,21]. These cells can be sub-divided into CD4^+^ and CD4^−^ (most of these CD4^−^CD8^−^) cells. CD4^−^CD8^−^ iNKT cells produce predominantly T-helper (Th)1 cytokines (IFNγ and TNFα) whereas CD4^+^ iNKT cells can produce both Th1 and Th2 (IL-4 and IL-13) cytokines [13]. Because of their unique capacity to rapidly produce large quantities of both Th1 (IFNγ) and Th2 (IL-4) cytokines upon stimulation [22], iNKT cells may have a key role in protection against tumors or in preventing autoimmune disease [23]. Despite low numbers, iNKT cells have a central role in intestinal homeostasis [17,24,25] and are essential for the development of oral tolerance [26,27]. Nevertheless, their number within the intraepithelial and lamina propria compartments and their specific role in CD pathogenesis remains elusive.

In this manuscript, we aimed to study whether changes in the number of iNKT cells may be altered in the duodenum of CD patients. To these aim we assessed the mRNA expression of Vα24-Jα18 and the proportion of iNKT cells within the intraepithelial compartment to reveal an increased number of these cells in the CD mucosa.

## 2. Materials and Methods

### 2.1. Patients and Biopsy Samples

Duodenal samples were collected from two independent populations in Spain (Hospital Clínico Universitario de Valladolid) and Argentina (Biobank from the LISIN, La Plata). The Spanish population included 25 untreated celiac patients (uCD, mean age 28.9 years; range 5–76 years; 42% males) (Table S1), 15 CD patients treated with GFD (GFD-CD; mean age 34.2 years; range 4-71 years; 34% males) (Table S2), 15 non-CD patients with other inflamed conditions (I-controls, mean age 42.1 years; range 15–78 years; 56% males) (Table S3) and 25 non-inflamed non-CD controls (C-controls; mean age 38.3 years; range 6–81 years; 30% males) (Table S4). The Argentinian population included 20 uCD patients (mean age 24.8 years; range 4-56 years; 28% males) (Table S5) and 19 C-controls (mean age 31.4 years; range 6–62 years; 52% males) (Table S6). Regarding age and gender, no statistically differences were found between Spanish and Argentinian patients. Clinical data from patient groups included in the study are shown in Table 1. The experiments were conducted with the understanding and the written consent of the adult participants, or the next of kin, caretakers, or guardians on behalf of the minors/children enrolled in this study. The study and the written consent procedure were approved by the Ethics committees from Hospital Clínico Universitario of Valladolid and Biobank from the LISIN, La Plata.

**Table 1:** Clinical data from patient groups included in the study.

	Study Patients	*n*	Mean age (Range)	Gender	HLA DQ2/DQ8	IgA anti- tTG/EMA	Marsh Criteria at Diagnosis	GFD
**Celiac Patients**	uCD	45	27.1 (4–76)	36% males	+	+	II-III	No
	GFD-CD	15	34.2 (4–71)	34% males	+	-	II-III	Yes
**Non Celiac Patients**	I-controls	15	42.1 (15–78)	56% males	+/-	- (*^A^)	0-I (M.M)	No
	C-controls	44	35.3 (6–81)	41% males	- (*^B^)	- (*^A^)	0	No

uCD (untreated celiac patients), GFD-CD (celiac patients treated with gluten free diet), I-controls (non celiac patients with other inflamed conditions) and C-controls (non-celiac disease patients without other inflamed conditions). IgA anti-tTG (Anti-tissue transglutaminase antibodies), EMA (Endomysium antibodies), GFD (Gluten free diet). M.M (mild mucosal alterations non compatibles with celiac disease). *^A^ Serological test were performed only in genetically susceptible patients. *^B^ Two patients had positive genetic susceptibility markers.

At diagnosis, all CD patients had CD-compatible symptoms, positive anti-endomysium and/or anti-transglutaminase IgA antibodies, CD-associated risk alleles (HLA-DQ2 and DQ8), and duodenal biopsy with histopathological changes. No differences in clinical markers were found between Spanish and Argentinian CD individuals. Patients on a GFD showed an improvement of the histological lesion (Marsh 0-I), and negative serum anti-transglutaminase antibodies for at least one year. Control groups were collected from patients referred to the gastroenterology clinics for diagnostic investigations due to clinical suspicion of intestinal disease (chronic diarrhea, gastritis by Helicobacter pylori, hiatus hernia, *etc.*). Similar symptoms were observed in both populations. Some of these cases showed duodenal inflammation (I-controls) while lack of mucosal affection was found in C-controls. None of them had a final diagnosis of CD.

### 2.2. Quantitative PCR

Duodenal biopsies from the Spanish (40 CD patients (25 uCD and 15 GFD-CD), 25 C-controls and 15 I-controls) and the Argentinian population (20 uCD and 19 C-controls) were submerged in 0.5 mL of RNALater^®^ solution (Ambion Inc, Austin, Texas, USA) and stored at −20 °C immediately after sample taking. Total RNA was isolated using the TRI-Reagent^®^ Solution according to manufacturer instructions (Ambion Inc, Austin, Texas, USA). In parallel, 15 duodenal samples from each group of patients from the Spanish population were also analyzed by flow cytometry as described below to determine the phenotype of lymphocytes and iNKT cells.

Reverse transcription was carried out by using the SuperScript^®^ First-Strand Synthesis System for reverse transcriptase (RT)-PCR Kit (Applied Biosystems, Carlsbad, CA, USA) with random hexamers as primers. Reactions were performed using the FastStart SYBR Green MasterMix (Roche Applied Science, Mannheim, Germany) with thermolabile Uracil DNA Glycosylase to prevent carry-over contamination. Messenger RNA levels (βactin, IFNγ, Vα24-Jα18 and FoxP3) were measured by quantitative PCR (qPCR) on a LightCycler^®^ instrument (Roche Applied Science, Mannheim, Germany) after extrapolation to an external curve. Primer sets and PCR conditions are described in Table 2. Levels of mRNA are expressed as the ratio molecule/βactin in arbitrary units (AU).

**Table 2:** Primer sequences for quantitative-PCR.

Molecule	Primers Sequence	NCBI Locus	Annealing T
βactin	fw: 5′ - ATG GGT CAG AAG GAT TCC TAT GTG - 3′ rv: 5′ - CTT CAT GAG GTA GTC AGT CAG GTC - 3′	NM_001101.3	60
IFNγ	fw: 5′ - TGG AAA GAG GAG AGT GAC AG - 3′ rv: 5′ - ATT CAT GTC TTC CTT GAT GG - 3′	NM_000619.2	60
Vα24-Jα18	fw: 5′ - CTG GAG GGA AAG AAC TGC - 3′ rv: 5′ - TGT CAG GGA AAC AGG ACC - 3′	NC_000014.9	65
FoxP3	fw: 5′ - CAG CAC ATT CCC AGA GTT CCT C - 3′ rv: 5′ - GCG TGT GAA CCA GTG GTA GAT C - 3′	NM_014009.3	60

Primer sequences used for quantitative-PCR. NCBI locus and annealing temperature point (annealing T). IFNγ (interferon-γ), Vα24-Jα18 (*invariant* TCRα chain of human iNKT cells), FoxP3 (intracellular transcription factor Forkhead Box P3).

### 2.3. Isolation of Intraepithelial Lymphocytes and Lamina Propria Mononuclear Cells

Biopsy samples from 15 uCD, 15 GFD, 15 non-inflamed non-CD controls (C-controls) and 15 inflamed non-CD controls (I-controls) were collected from the Spanish population. Samples were kept in ice-chilled physiologic phosphate buffered saline (PBS) and processed within an hour as previously described [28,29]. Briefly, IEL and epithelial cells were released from the mucosal specimens by incubation for 1 hour under gentle agitation with 1 mM ethylenediaminetetraacetic acid (EDTA) and 1 mM dithiothreitol (DTT) in RPMI 1640 medium (GibcoBRL Life Technologies, Vienna, Austria) supplemented with 10% fetal calf serum, 2mM l-glutamine, 100U/mL penicillin, 100 μg/mL streptomycin and 0.25 μg/mL amphotericin (GibcoBRL Life Technologies, Vienna, Austria). Following DTT and EDTA incubation IEL were released into the medium and collected by centrifugation, washed twice in PBS (Lonza, Braine-l’Alleud, Belgium) and stained with fluorochrome-conjugated monoclonal antibodies (mAbs).

The remaining tissue was incubated in moderated rotation at 37 °C for 90–120 min. with 1 mg/mL of collagenase D in RPMI 1640 medium (GibcoBRL Life Technologies, Vienna, Austria) supplemented with 10% fetal calf serum and antibiotics until the biopsies have been completely degraded. Single cell suspensions were filtered (70 μm Nylon Filter, BD Biosciences, San Diego, CA, USA) to remove non-cellular fibers, and the lamina propria mononuclear cells (LPMC) suspension was washed twice in PBS.

### 2.4. Antibody Labeling and Flow Cytometry Analysis

A total of 100,000 isolated cells (IEL or LPMC) were labeled with fluorochrome-conjugated monoclonal antibodies (mAbs) and their appropriate isotype-matched control antibodies from the same manufacturers. The fluorochrome-conjugated mAbs were: FITC Mouse anti-human CD103 (clone Ber-ACT8), PE Mouse anti-human Vα24-Jα18 (clone 6B11, specifically recognizing all T cells expressing the conserved CDR3 region of the Vα24Jα18 invariant TCRα rearrangement), PE Mouse anti-human TCRγδ (clone B1), APC Mouse anti-human CD3 (clone HIT3a), APC Mouse anti-human CD25 (clone M-A251), PE-Cy7 Mouse anti-human CD8 (clone RPA-T8) and PE-Cy7 Mouse anti-human CD45 (clone HI30) from BD Pharmingen (San Diego, CA, USA); PE Mouse anti-human FoxP3 (clone PCH101) from eBioscience (San Diego, CA, USA); FITC Mouse anti-human CD4 (clone 13B8.2) and PE Mouse anti-human CD8 (clone B9.11) from Beckman Coulter (Brea , CA, USA). Cells were labeled in phosphate-buffered saline containing 1 mM EDTA and 0.02% sodium azide (fluorescent-activated cell sorting (FACS) buffer). Labeling was performed on ice and in the dark for 20 min. Cells were washed twice in FACS buffer, fixed with 1% paraformaldehyde in 0.85% saline, and stored at 4 °C before acquisition on the flow cytometer within 24 h. For FoxP3 intracellular staining, cells were fixed with Leucoperm A following surface staining and permeabilized with Leucoperm B (Bio-Rad, UK) before adding antibody for intracellular labeling. After incubation cells were washed in FACS buffer, fixed, and acquired as previously reported.

**Figure 1:**
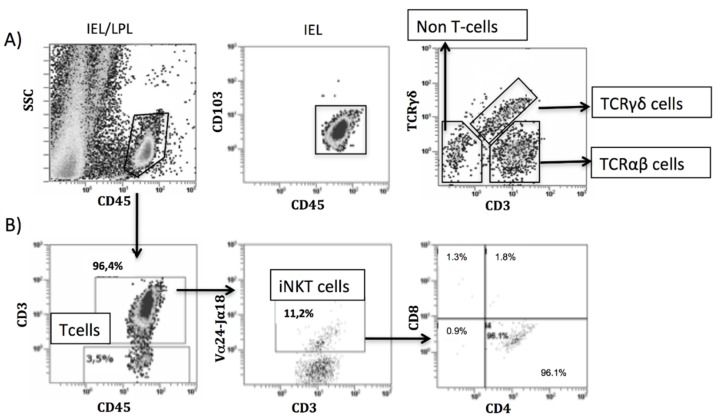
Identification of intraepithelial and lamina propria lymphocytes by flow cytometry. Example of characterization of intraepithelial lymphocytes (IELs)/lamina propria lymphocytes (LPL) in an untreated celiac disease donor. Lamina propria (CD45^+^) and IEL (CD45^+^CD103^+^) were identified and percentages of TCRαβ cells, TCRγδ cells and non-T cells determined (**A**). Example of characterization of invariant NKT (iNKT) cells in an untreated celiac disease donor: CD45^+^CD3^+^Vα24-Jα18^+^ cells within the total of CD45^+^CD3^+^ cells, iNKT (CD45^+^CD3^+^Vα24-Jα18^+^) phenotype according to the expression of CD4 and/or CD8 within the total of iNKTs (**B**).

Cells were acquired in a Beckman Coulter FC500 flow cytometer and data processed with Cell BC software (Beckman Coulter, Brea, CA, USA). All IEL and lamina propria lymphocyte (LPL) cells were identified as CD45^+^ (leukocyte pan-marker) and IELs were also identified as CD103^+^. Non-T cells (CD3^−^), TCRγδ cells (CD3^+^TCRγδ^+^), TCRαβ cells (CD3^+^TCRγδ^−^) (Figure 1A), iNKT cells (CD3^+^Vα24-Jα18^+^) (Figure 1B) and Treg cells (CD3^+^CD4^+^CD25^+^FoxP3^+^ or CD3^+^CD4^+^FoxP3^+^) were identified by flow cytometry within the intraepithelial and the lamina propria compartments. Numbers of cells were expressed as percentages.

### 2.5. Statistical Analysis

Correlation analyses and two-tailed non-parametric statistical analyses were performed using the Kruskal-Wallis one way analysis of variance test, the Mann-Whitney U test and the non-parametric Spearman’s correlation. *p* < 0.05 was considered significant. Flow cytometry results were expressed as percentages and analysed by the two-tailed non-parametric Mann-Whitney U test. *p* < 0.05 was considered significant. Reference values of the IEL subpopulations for each patient’s group were expressed as median percentages with the interquartile range (IQR).

## 3. Results

### 3.1. Increased Duodenal Vα24-Jα18 mRNA Expression in Celiac Disease Patients

Due to the low numbers of iNKT cells both in blood [21] and tissue samples [22] we first studied the proportion of iNKT cells in duodenal biopsies by assessing the mRNA expression or their restrictive invariant Vα24-Jα18 chain in complete biopsy explants [20,21].

**Figure 2:**
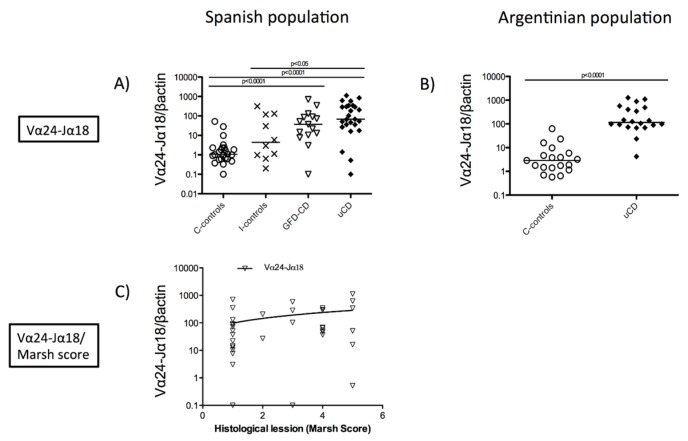
Increased duodenal Vα24-Jα18 mRNA expression in untreated and treated celiac disease patients. Expression levels of Vα24-Jα18 mRNA in duodenal biopsies indicated by the ratio Vα24-Jα18/βactin in arbitrary units (AU), in untreated Celiac Disease (uCD), Gluten Free Diet-CD patients (GFD-CD), inflamed non-CD controls (I-controls) and non-inflamed non-CD controls (C-controls) in the Spanish population (**A**) and in uCD and C-controls in the Argentinian population (**B**). Statistically significant differences are shown (two tailed Mann-Whitney U test; Krustall-Wallis test). Horizontal bars are median values. Correlation between the degree of histological lesion (Marsh score) and the expression level of Vα24-Jα18 mRNA in CD patients. (1: Marsh 0-I, 2: Marsh II, 3: Marsh IIIa, 4: Marsh IIIb, 5: Marsh IIIc) There was an increased of Vα24-Jα18 expression and the duodenal increased level of atrophy (Spearman *r* = 0.063, *p <* 0.05). GFD-CD patients show a Marsh 0-I, uCD show duodenal atrophy Marsh II to IIIc (**C**).

Untreated-CD patients (uCD) had increased Vα24-Jα18 mRNA levels compared to both inflamed non-CD (I-controls) (*p <* 0.05) and non-inflamed non-CD controls (C-controls) (*p <* 0.001) (Figure 2A). Gluten Free Diet-CD patients (GFD-CD) had increased Vα24-Jα18 mRNA levels compared to C-controls (*p <* 0.001). No differences were found between uCD and GFD-CD patients, which suggests an increased load of iNKT cells in duodenal biopsies from both groups of patients irrespectively of the disease status (Figure 2A). We also analyzed duodenal expression of Vα24-Jα18 mRNA in an independent population from Argentina. Figure 2B confirms that CD patients show increased duodenal Vα24-Jα18 mRNA levels compared to non-inflamed non-CD controls (*p <* 0.001), irrespectively of the origin of the samples. No statistically significant differences in Vα24-Jα18 mRNA levels were found between the Spanish and the Argentinian populations, neither within the control nor the untreated CD patient groups.

Samples from GFD-CD patients showed a Marsh score for the severity of the mucosal lesion between 0 and I, and no differences were found in these sub-groups regarding Vα24-Jα18 mRNA expression. Untreated CD patients had a Marsh score rating from I to IIIc, and in these patients, Vα24-Jα18 mRNA levels correlated with the Marsh score (Spearman’s *r* = 0.063, *p <* 0.05) (Figure 2C).

### 3.2. Correlation between Duodenal Vα24-Jα18 and IFNγ mRNA Expression in Celiac Disease

**Figure 3:**
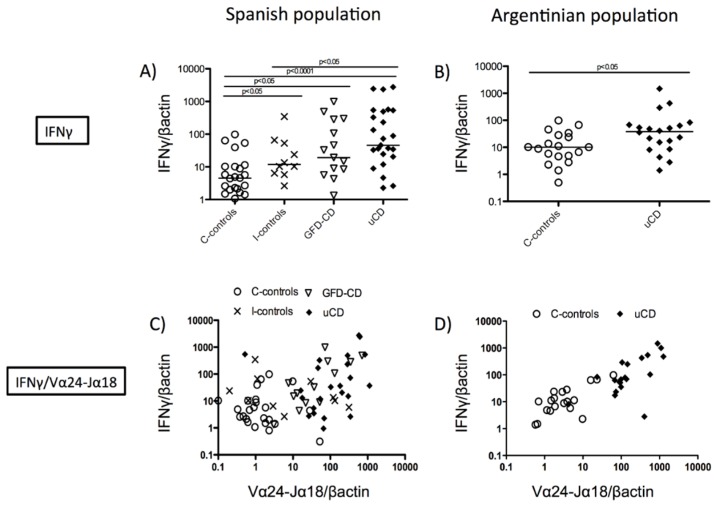
Correlation between duodenal mRNA expression of Vα24-Jα18 and IFNγ. Expression levels of IFNγ mRNA in duodenal biopsies indicated as the ratio IFNγ/βactin, in arbitrary units (AU), in untreated Celiac Disease (uCD), Gluten Free Diet-CD patients (GFD-CD), inflamed non-CD controls (I-controls) and non-inflamed non-CD controls (C-controls) in the Spanish population (**A**) and in uCD and C-controls in the Argentinian population (**B**). Statistically significant differences are shown (two tailed Mann-Whitney U test; Krustall-Wallis test). Horizontal bars are median values. Correlation between the expression of IFNγ and Vα24-Jα18 mRNA, in arbitrary units (AU) in the Spanish population (**C**) (C-controls: Spearman *r* = 0.3115, *p* value = n.s; I-controls: Spearman *r* = 0.4265, *p* value = n.s; GFD-CD: Spearman *r* = 0.5393, *p* value < 0.05; uCD: Spearman *r* = 0.4323, *p* value < 0.05) and in the Argentinian population (**D**) (C-controls: Spearman *r* = 0.5895, *p* value < 0.05; uCD: Spearman *r* = 0.6917, *p* value < 0.001).

Duodenal samples from CD patients (either treated and untreated) had increased IFNγ mRNA expression compared with non-inflamed non-CD controls both in the Spanish (GFD-CD, *p <* 0.05; uCD, *p <* 0.001) (Figure 3A) and the Argentinian populations (uCD, *p <* 0.05) (Figure 3B) as previously described [30,31]. Since both mRNA levels of IFNγ [2,30] and Vα24-Jα18 (Figure 2C) correlated with the Marsh score for the severity of the mucosal lesion, we studied if the expression of IFNγ and Vα24-Jα18 was related. A correlation was found between the mRNA levels of IFNγ and Vα24-Jα18 in treated and untreated CD samples in both the Spanish (C-controls: Spearman’s *r* = 0.3115, *p* value = n.s; I-controls: Spearman’s *r* = 0.4265, *p* value = n.s; GFD-CD: Spearman’s *r* = 0.5393, *p <* 0.05; uCD: Spearman’s *r* = 0.4323, *p <* 0.05) and the Argentinian populations (C-controls: Spearman’s *r* = 0.5895, *p <* 0.05; uCD: Spearman’s *r* = 0.6917, *p <* 0.001) (Figure 3C,D).

### 3.3. Duodenal Vα24-Jα18 and FoxP3 mRNA Levels Reveal a Celiac Disease Molecular Profile

**Figure 4:**
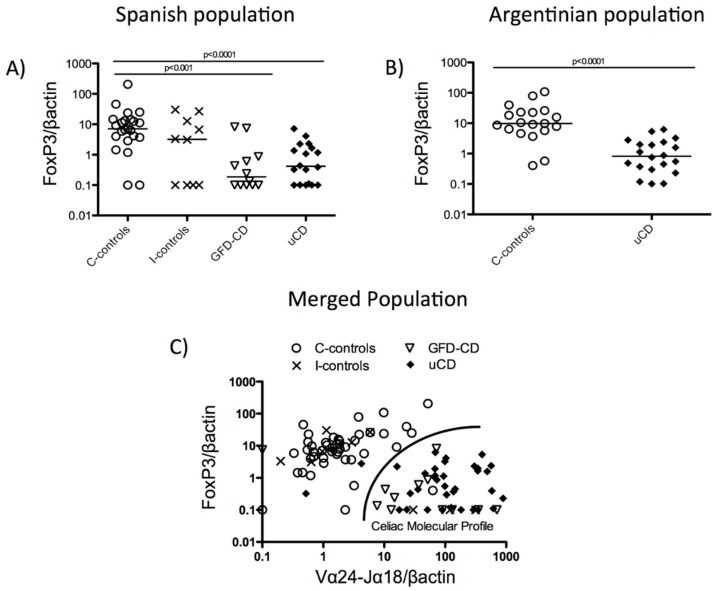
Duodenal mRNA levels of Vα24-Jα18 and FoxP3 reveal a celiac disease molecular profile. Expression levels of FoxP3 mRNA in duodenal biopsies indicated as the ratio FoxP3/βactin, in arbitrary units (AU) in untreated Celiac Disease (uCD), Gluten Free Diet-CD patients (GFD-CD), inflamed non-CD controls (I-controls) and non-inflamed non-CD controls (C-controls) in the Spanish population (**A**) and in uCD and C-controls in the Argentinian population (**B**). Statistical differences are shown (two tailed Mann-Whitney U test; Krustall-Wallis test). Horizontal bars are median values. *Duodenal molecular profile*: Correlation between expression levels of FoxP3 and Vα24-Jα18 mRNA, in arbitrary units (AU) (C-controls: Spearman *r* = 0.3495, *p* value < 0.05; I-controls: Spearman *r* = −0.4577, *p* value = n.s; GFD-CD: Spearman *r* = −0.4858, *p* value < 0.05; uCD: Spearman *r* = −0.056, *p* value = n.s) (**C**).

We next studied FoxP3 mRNA expression in the duodenum as an indirect way of quantifying Treg cells in tissue. CD samples (both uCD and GFD-CD) had decreased FoxP3 expression compared with non-inflamed non-CD-controls (C-controls) (uCD, *p <* 0.001; GFD-CD, *p <* 0.001) (Figure 4A). Similar results were found on a second independent analysis of the Argentinian population (uCD, *p <* 0.001) (Figure 4B). Because CD samples were characterized by increased duodenal mRNA expression of Vα24-Jα18 (Figure 2A,B) and decreased expression of FoxP3 (Figure 4A,B), we studied whether the joint analysis of these two molecules could help us to identify a CD-like molecular profile.

No differences were found between samples from controls or uCD patients in any of the molecules studied in both populations (Spanish and Argentinian). Similar results were also obtained when Vα24-Jα18 and FoxP3 duodenal mRNA expression were independently analysed. Therefore, both populations were merged to increase the sample size of uCD and C-control groups. The joint analysis of both duodenal FoxP3 and Vα24-Jα18 mRNA levels allowed us to discriminate between CD (treated and untreated), and non-CD samples (inflamed or non-inflamed) with a sensibility and sensitivity of 92%, revealing a CD-like molecular profile (C-controls: Spearman’s *r* = 0.3495, *p <* 0.05; I-controls: Spearman’s *r* = −0.4577, *p* value = n.s; GFD-CD: Spearman’s *r* = −0.4858, *p <* 0.05; uCD: Spearman’s *r* = −0.056, *p* value = n.s) (Figure 4C).

All together, our findings suggest an increase of iNKT cells in the CD duodenum as determined by molecular approaches. Therefore, we next studied the number of iNKT cells within the intraepithelial and lamina propria compartments by flow cytometry. To that end, the IEL profile was first analyzed and compared with previous reports before determining the number of iNKT cells in the CD duodenum.

### 3.4. Intraepithelial Lymphocytes in the Duodenum from Celiac Disease Patients

Total IELs (CD103^+^CD45^+^), non-T cells (CD103^+^CD45^+^CD3^−^), TCRγδ^+^ cells (CD103^+^CD45^+^CD3^+^TCRγδ^+^) and TCRαβ^+^ cells (CD103^+^CD45^+^CD3^+^TCRγδ^−^) were studied within the intraepithelial compartment (as characterized in Figure 1A) given their relevance as biomarkers in CD diagnosis [9,32].

**Figure 5:**
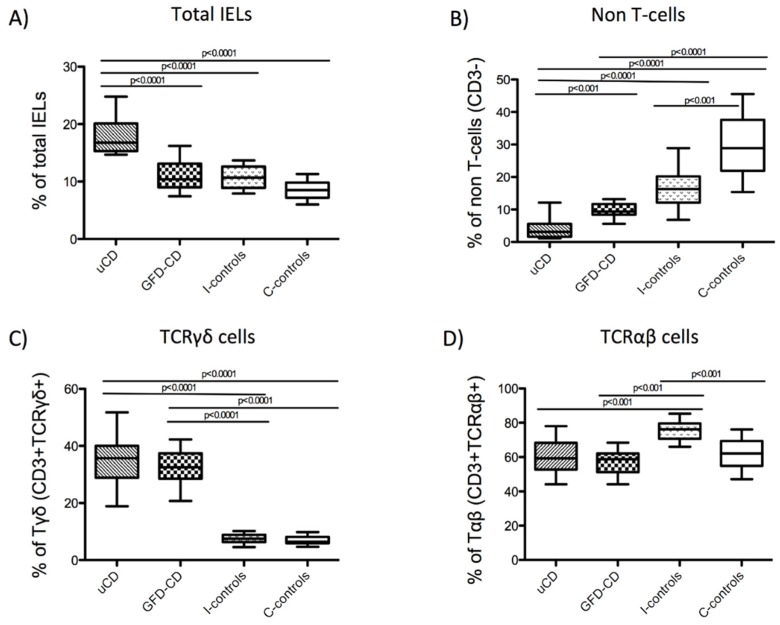

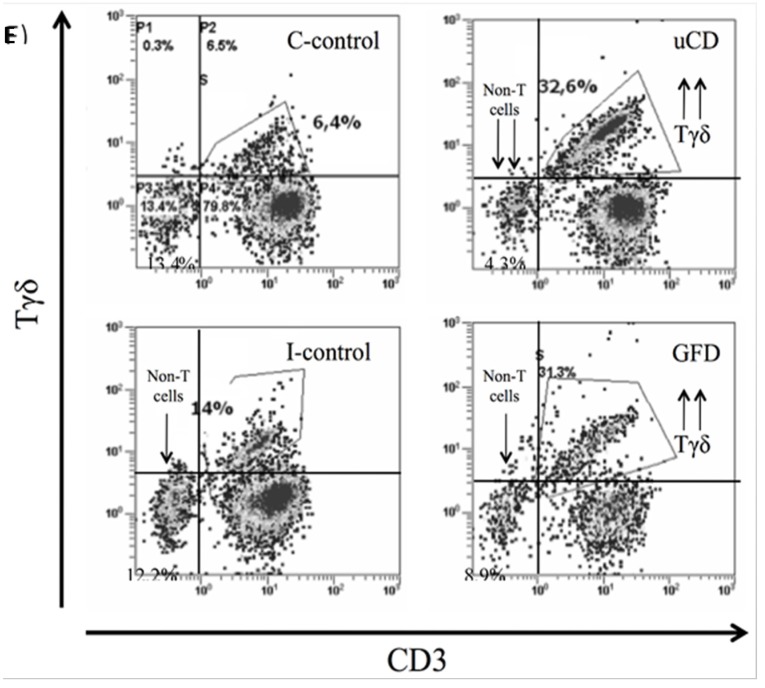
Phenotype of intraepithelial lymphocytes in the duodenum from celiac patients. Phenotype of IELs in untreated Celiac Disease (uCD), Gluten Free Diet-CD patients (GFD-CD), inflamed non-CD controls (I-controls) and non-inflamed non-CD controls (C-controls), analyzed by flow cytometry: Percentage of total IELs (CD103^+^CD45^+^) referred to the total of epithelial cells (**A**). Percentage of non-T cells (CD103^+^CD45^+^CD3^−^) (**B**), Tγδ cells (CD103^+^CD45^+^CD3^+^TCRγδ^+^) (**C**) and Tαβ cells (CD103^+^CD45^+^CD3^+^TCRγδ^−^) (**D**) referred to the total of IELs. Horizontal bar are median values. Statistically significant differences are shown (two tailed Mann Whitney U test; *p <* 0.05). Representative flow cytometry analysis data of IEL subpopulations (Tγδ, Tαβ and non-T cells) in uCD, GFD-CD, I-controls and C-controls (**E**).

Untreated CD patients had increased numbers of total IELs (median/IQR; 16.80%/4.80) (Figure 5A,E) together with decreased numbers of non-T cells (3.10%/4.00) (Figure 5B,E). The latter was also true in GFD-CD patients although the total number of IELs did not increase (GFD-CD, 10.40%/4.11) (Figure 5A,E). Within the CD3^+^ subpopulation, both uCD and GFD-CD patients showed higher numbers of TCRγδ^+^ cells (uCD, 35.78%/11.13; GFD-CD, 32.50%/8.90) (Figure 5C,E), previously described as a distinctive feature of CD patients [9,32]. In support of this, inflamed non-CD controls did not have increased numbers of TCRγδ^+^ cells (I-controls, 7.51%/2.60) (Figure 5C,E) despite the decreased percentage of non-T cells compared with non-inflamed non-CD controls (I-controls, 16.30%/8.12; C-controls, 28.90%/15.75) (Figure 5B,E). However, these patients had increased numbers of TCRαβ^+^ cells (I-controls, 76.20%/8.20) compared with the remainder patient groups (uCD, 59.30%/15.6; GFD-CD, 58.80%/10.90; C-controls, 62.10%/14.5) (Figure 5D,E).

Within the intraepithelial compartment, a decreased number of non-T cells together with increased TCRγδ^+^ cells represent a distinctive pattern of CD patients irrespectively of the disease status [32,33]. Reference values of the IEL subpopulations for each patient’s group were expressed as median percentages with the interquartile range (IQR) in Table 3.

**Table 3:** Specificity of intraepithelial lymphocytes profiling in the diagnosis of celiac disease.

	uCD Median (IQR)	GFD-CD Median (IQR)	I-controls Median (IQR)	C-controls Median (IQR)
**Total IELs**	16.80% (4.80)	10.40% (4.11)	10.70% (3.70)	8.50% (2.60)
**Non-T cells**	3.10% (4.00)	9.34% (3.34)	16.30% (8.12)	28.90% (15.75)
**TCRγδ^+^cells**	35.78% (11.13)	32.50% (8.90)	7.51% (2.60)	6.44% (2.38)
**TCRαβ^+^cells**	59.30% (15,6)	58.80% (10,9)	76.20% (8,9)	62.10% (14,5)

Percentages of duodenal Intraepithelial lymphocyte (IEL) populations in untreated Celiac Disease (uCD), Gluten Free Diet-CD patients (GFD-CD), inflamed non-CD controls (I-controls) and non-inflamed non-CD controls (C-controls) expressed as median percentages with the interquartile range (IQR).

After characterizing the duodenal IEL profile in CD patients and controls, we finally assessed whether the percentage of iNKT cells was increased in the CD duodenum, as suggested by molecular studies.

### 3.5. Increased Intraepithelial iNKT Cells in Celiac Disease Patients and Correlation with Vα24-Jα18 mRNA Expression

**Figure 6:**
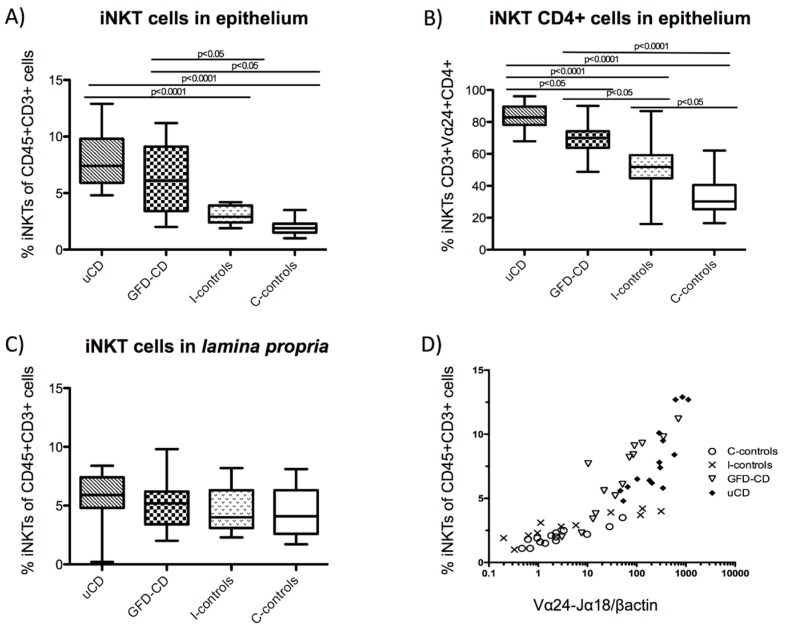
Increased intraepithelial iNKT cells in celiac disease patients and correlation with Vα24-Jα18 mRNA expression. Percentage of iNKT cells (CD45^+^CD3^+^Vα24-Jα18^+^) among the total number of Intraepithelial Lymphocytes (IELs) in untreated Celiac Disease (uCD), Gluten Free Diet-CD patients (GFD-CD), inflamed non-CD controls (I-controls) and non-inflamed non-CD controls (C-controls) (**A**). Percentage of Intraepithelial CD4^+^ iNKT cells among the total number of iNKT cells in aCD, GFD-CD, I-controls and C-controls (**B**). Percentage of iNKT cells among the total number of Lamina Propria Lymphocytes (LPLs) in aCD, GFD-CD, I-controls and C controls (**C**). Horizontal bars are median values. Statistically significant differences are shown (two tailed Mann Whitney U test; *p <* 0.05). Correlation between Vα24-Jα18 mRNA expression, in arbitrary units (AU) and the percentage of iNKT cells among the total number of IELs in aCD, GFD-CD, I-controls and C controls (C-controls: Spearman *r* = 0.8603, *p* value<0.001; I-controls: Spearman *r* = −0.9455, *p* value < 0.0001; GFD-CD: Spearman *r* = −0.9297, *p* value < 0.0001; uCD: Spearman *r* = 0.8287, *p* value = 0.001) (**D**).

As suggested by molecular studies (Figure 2A,B), iNKT cells (as characterized in Figure 1B) were increased within the IEL compartment from CD patients (in both, uCD: 7.4%/3.9 and GFD-CD: 6.1%/5.7) compared with non-CD groups (C-controls: 1.9%/0.8 and I-controls: 2.9%/1.5) (Figure 6A).

There were also differences in the phenotype of intraepithelial iNKT cells since CD patients had a higher proportion of CD4^+^ iNKT cells (uCD: 82.9%/11.4, GFD-CD: 70.0%/10.5, I-controls: 51.8%/14.5, C-controls: 30.2%/15.1) (Figure 6B). However, the number of iNKT cells from the lamina propria did not differ in number in any of the groups (Figure 6C).

As previously shown, the mRNA expression of Vα24-Jα18 was analyzed by qPCR in all of these duodenal biopsies (Figure 2A). For that reason, we studied whether the increased Vα24-Jα18 mRNA expression could be used as a marker of the increased number of intraepithelial iNKT cells found by flow cytometry (Figure 6A). As shown in Figure 6D, the percentage of iNKT cells correlated with Vα24-Jα18 mRNA levels (C-controls: Spearman’s *r* = 0.8603, *p <* 0.001; I-controls: Spearman’s *r* = −0.9455, *p <* 0.001; GFD-CD: Spearman’s *r* = −0.9297, *p <* 0.001; uCD: Spearman’s *r* = 0.8287, *p* = 0.001), which confirms our findings, but also that the study of Vα24-Jα18 mRNA levels may be a valid approach to characterize the density of iNKT cells in complex tissues.

## 4. Discussion

Despite their low numbers, iNKT cells may have an essential role for the immune homeostasis in the gastrointestinal tract [17,34]. Here, we studied intestinal iNKT cells in the context of celiac disease by molecular and cellular approaches, and found higher numbers of both total and CD4^+^ iNKT cells in the intraepithelial compartment of CD patients. We also found a correlation between the mRNA expression of Vα24-Jα18 and (i) the severity of the mucosal lesion, and (ii) the mRNA expression of IFNγ. Finally, the mRNA expression of both Vα24-Jα18 and FoxP3 might define an mRNA CD-like molecular profile. Altogether our findings suggest that the number of duodenal intraepithelial iNKT cells is increased in CD patients.

Invariant NKT cells have a key role in the mechanism of oral tolerance [26,34], and their hepatic depletion leads to the inability of developing oral tolerance in a mouse model [35]. These cells may have an effect in the development of tolerogenic dendritic cells, which are responsible for the proliferation of regulatory T cells [13]. Because of the innate and adaptive features of these cells, and the ability to produce high levels of IL-4 and IFNγ [17], a role for iNKT cells has been suggested in inflammatory bowel disease [36] and in CD [37]. Some studies have characterized circulating numbers of iNKT cells in CD patients, often with contradictory results [21,37,38], but few have reported the number of these cells in the duodenum [37,39,40].

Profiling IEL subpopulations has been used as a tool in the diagnostic work out of CD [9,32,41,42]. Using flow cytometry, a method previously validated by Camarero *et al*. [5,9], we have found similar percentages of IEL subpopulations than previous reports using similar [9,32] and different methods [43], therefore reassuring that we have successfully identified iNKT within IELs. However, these results are opposed to those from Calleja *et al.* [39] who did not find increased total IELs, as it has been previously described [9]. Moreover, neither Calleja *et al.* [39] nor Dunne *et al.* [40] found an increased numbers of iNKT cells in the intraepithelial compartment of CD patients using a similar method. Unfortunately, the nature of these differences between the results reported by these authors and our study remains elusive. However, our finding of a strong correlation between iNKT cells within the intraepithelial compartment and tissue mRNA expression of Vα24-Jα18 confirms a higher density of iNKT cells in the CD duodenum.

A very interesting question is whether the increased numbers of iNKT cells found in the intraepithelial compartment of these patients correlates with similar changes in the number of circulating iNKT cells. However, the study of circulating iNKT cells was not the aim of this research and hence blood samples had not been obtained from patients as we had performed in a previous study [21]. Future analysis will address this issue including the characterization (e.g., homing profile) of circulating iNKT cells in CD patients.

Grose *et al*. consistently reported reduced numbers of intestinal iNKT cells in CD as determined by qPCR [37,44] and by immunofluorescence [37], although in the later study the authors did not discriminate between intraepithelial and lamina propria cells [37]. However, we found increased numbers of iNKT cells in the intraepithelial compartment, but not in the lamina propria. Our findings were also correlated with mRNA expression levels of Vα24-Jα18 despite our qPCR results are not in agreement with the former studies [37,44]. Such discrepancy might be explained because duodenal Vα24-Jα18 mRNA expression varies between different populations. Our results have been confirmed in two different sets of samples from individuals from different continents. Besides, the increased Vα24-Jα18 mRNA expression found in the duodenum of CD patients also correlated with the percentage of intraepithelial iNKT cells by flow cytometry, with the Marsh score for the severity of the lesion, and with the mRNA expression of IFNγ, giving further consistency to our results. We are aware that the expression of the TCR Vα24 chain is not exclusive of iNKT cells and therefore we may be identifying other cell types (by flow cytometry and qPCR).However, the analysis of the Vα24-Jα18 molecule is more restrictive than Vβ11 [21,38]. In fact, the identification of iNKT cells by mRNA expression has been recently proposed as a more reliable alternative than the previously used co-expression of CD3^+^ and CD161^+^, because T cells (non-NKT) may induce CD161^+^ expression after activation [45]. Moreover, we have found a direct correlation between Vα24-Jα18 mRNA expression and the total numbers of Vα24-Jα18^+^ cells (representative of pure iNKT cells) in the intraepithelial compartment as determined by flow cytometry. Therefore, we are confident that Vα24-Jα18 mRNA expression is representative of the total numbers of iNKT cells in the duodenal mucosa.

Intraepithelial lymphocytes are an heterogeneous population of T cells and non-T cells, mainly composed of cytotoxic CD8^+^ T cells, whose main role is the maintenance of the epithelial integrity by eliminating stressed cells and promoting epithelial repair [6]. Some authors have tried to identify the nature of non-T cells within the IEL compartment, which have been characterized as mainly NK cells, but also T cell precursors [46,47]. Dysregulated activation and increased IEL T cell numbers is a hallmark of CD and this is critically involved in epithelial cell destruction and subsequent development of villous atrophy [7,10]. The mechanisms underlying the massive expansion of IFNγ–producing intraepithelial cytotoxic T lymphocytes (CTLs) and the destruction of the epithelial cells lining the small intestine of CD patients is the focus of current research. Meresse *et al*. [10] reported an oligoclonal expansion of CTLs in CD that exhibits profound genetic reprogramming of NK cell functions. These CTLs expressed aberrant cytolytic NK lineage receptors, such as NKG2C, NKp44, and NKp46, which associated with adaptor molecules bearing immunoreceptor tyrosine-based activation motifs, induce ZAP-70 phosphorylation, cytokine secretion, and proliferation independently of TCR signalling as well as downregulation of the TCR. All of these features are characteristic of the iNKT population [48,49] though we cannot conclude that they really perform these functions in the CD duodenum and are responsible of tissue damage in CD.

IFNγ is mainly produced by the gluten-specific Th1 cells and is essential in CD pathogenesis. Recent studies also suggest that IELs are an important source of IFNγ, and the production of IFNγ persists even after GFD [8]. Our results show a correlation between the increased mRNA expression of IFNγ in CD patients (both treated and untreated) and the mRNA expression of Vα24-Jα18. These results together with the correlation between the mRNA expression of Vα24-Jα18, the Marsh score for the severity of the mucosal lesion and the total number of intraepithelial iNKT cells might suggest that the increased of IFNγ in the CD duodenum might be favoring an increased recruitment of iNKT cells. However, this requires further studies, which may be difficult to perform given the low number of iNKT cells. At present, it is considered that the natural ligands of iNKT cells are glycolipids from the cytoplasm of enterocytes, released to the extracellular matrix after apoptosis or necrosis [18,50,51], favoured by an environment rich in IFNγ and IL-15 characteristic in CD [52,53], and IL-15 plays a central role in the biological function of iNKT cells [54]. This proinflammatory environment may be relevant for the increase and activation of intraepithelial iNKT cells which, in turn, may be also a source of IFNγ.

We also studied FoxP3 mRNA expression as an indirect measurement of Treg and found a lower expression in the CD duodenum. However, we were unable to identify T cells expressing intracellular FoxP3 neither in the duodenal lamina propria nor in the intraepithelial compartment by flow cytometry (data not shown). A possible explanation might be that FoxP3 expression in humans is transient, dependent on the environment and not restricted to T-cells with a *Treg* phenotype [55,56]. In addition, and opposed to murine models [57], there is evidence suggesting that FoxP3 can be also expressed in cells without regulatory function, such as epithelial and tumor cells [58,59,60]. Therefore it is likely that we were not identifying T cells expressing FoxP3 by qPCR but other non-T cell types which have remained elusive by flow cytometry. Nonetheless, a CD-like molecular profile based on the mRNA expression of Vα24-Jα18 and FoxP3, which is also observed in GFD-CD patients, might be used as a useful diagnostic biomarker.

In conclusion, we have found an increased number of iNKT cells in the duodenum from both uCD and GFD-CD patients, irrespective of the mucosal status. Increased mRNA levels of Vα24-Jα18 correlated with the severity of the mucosal lesion and with the mRNA levels of IFNγ. A CD-like molecular profile, defined by an increased mRNA expression of Vα24-Jα18 together with a decreased expression of FoxP3, may represent a pro-inflammatory signature of the CD duodenum.

## Supplementary Materials

Supplementary materials can be accessed at: http://www.mdpi.com/2072-6643/7/11/5444/s1.
