# Supplementary material for: Increased Intraepithelial Vα24 Invariant NKT Cells in the Celiac Duodenum
Source: Nutrients. 2015 Oct 30;7(11):8960–76. doi: 10.3390/nu7115444 (PMC4663572; doi:10.3390/nu7115444)
Supplement: Supplementary file 1 [file nutrients-07-05444-s001.docx]

Supplementary Material

**Table S1.** Clinical data from untreated Celiac Disease patients (uCD, *n* = 25) from the Spanish population.

| **Sample** | **Gender** | **Age** | **HLA DQ2/DQ8** | **IgA**  **Anti-tTG** | **Marsh Criteria at Diagnosis** |
| --- | --- | --- | --- | --- | --- |
| uCD 1 | M | 12 | + | + | III |
| uCD 2 | M | 34 | + | + | III |
| uCD 3 | F | 25 | + | + | III |
| uCD 4 | F | 32 | + | + | III |
| uCD 5 | F | 62 | + | + | III |
| uCD 6 | M | 10 | + | + | II |
| uCD 7 | F | 11 | + | + | III |
| uCD 8 | M | 7 | + | + | M.M |
| uCD 9 | F | 5 | + | + | III |
| uCD 10 | F | 48 | + | + | III |
| uCD 11 | F | 28 | + | + | III |
| uCD 12 | F | 11 | + | + | I |
| uCD 13 | M | 55 | + | + | III |
| uCD 14 | F | 27 | + | + | III |
| uCD 15 | F | 12 | + | + | N.D |
| uCD 16 | M | 43 | + | + | III |
| uCD 17 | F | 72 | + | + | III |
| uCD 18 | F | 16 | + | + | III |
| uCD 19 | M | 42 | + | - (EMA+) | III |
| uCD 20 | F | 76 | + | + | III |
| uCD 21 | M | 21 | + | + | III |
| uCD 22 | M | 11 | + | + | III |
| uCD 23 | F | 9 | + | + | III |
| uCD 24 | F | 24 | + | + | N.D |
| uCD 25 | M | 29 | + | + | III |

Table shows the following information: gender (M: male and F: female), age (from 5 to 76 years), HLA DQ2/DQ8, IgA anti tTG (Anti-tissue transglutaminase antibodies) and Marsh scale of the severity of lesion at diagnosis (from Marsh I to Marsh III). N.D. (Not determined). M.M (mild mucosal alterations). EMA (Endomysium antibodies)

**Table S2.** Clinical data from Celiac patients on a Gluten-Free Diet (GFD-CD, *n* = 15) from the Spanish population.

| **Sample** | **Gender** | **Age** | **HLA DQ2/DQ8** | **IgA**  **Anti-tTG** | **Marsh Criteria at Diagnosis** | **Years on GFD** |
| --- | --- | --- | --- | --- | --- | --- |
| GFD-CD 1 | F | 61 | + | - | III | 3 |
| GFD-CD 2 | M | 21 | + | - | III | 2 |
| GFD-CD 3 | F | 24 | + | - | II | 1 |
| GFD-CD 4 | M | 34 | + | - | III | 9 |
| GFD-CD 5 | F | 33 | + | - | III | 10 |
| GFD-CD 6 | F | 14 | + | - | III | 7 |
| GFD-CD 7 | F | 4 | + | - | III | 1 |
| GFD-CD 8 | M | 27 | + | - | N.D | >10 |

**Table S2.** *Cont.*

| **Sample** | **Gender** | **Age** | **HLA DQ2/DQ8** | **IgA**  **Anti-tTG** | **Marsh Criteria at Diagnosis** | **Years on GFD** |
| --- | --- | --- | --- | --- | --- | --- |
| GFD-CD 9 | F | 42 | + | - | III | >10 |
| GFD-CD 10 | M | 15 | + | - | II | 6 |
| GFD-CD 11 | F | 51 | + | - | III | >10 |
| GFD-CD 12 | F | 68 | + | - | I | >10 |
| GFD-CD 13 | F | 40 | + | - | III | 2 |
| GFD-CD 14 | M | 71 | + | - | III | >10 |
| GFD-CD 15 | F | 9 | + | - | M.M | 2 |

Table shows the following information: gender (M: male and F: female), age (from 4 to 71 years), HLA DQ2/DQ8, IgA anti tTG (Anti-tissue transglutaminase antibodies), Marsh scale of the severity of lesion at diagnosis (from Marsh I to Marsh III) and years on Gluten-Free Diet (GFD). N.D. (Not determined). M.M (mild mucosal alterations).

**Table S3.** Clinical data from non-celiac disease patients with other inflamed conditions (I-controls,
*n* = 15) from the Spanish population.

| **Sample** | **Gender** | **Age** | **HLA DQ2/DQ8** | **IgA**  **Anti-tTG** | **Marsh Criteria at Diagnosis** | **GFD** |
| --- | --- | --- | --- | --- | --- | --- |
| I-control 1 | M | 54 | - | - | 0 | No |
| I-control 2 | F | 36 | + | - | 0 | No |
| I-control 3 | F | 73 | + | - | I | No |
| I-control 4 | M | 15 | - | - | 0 | No |
| I-control 5 | M | 61 | - | - | 0 | No |
| I-control 6 | F | 46 | - | - | 0 | No |
| I-control 7 | M | 31 | - | - | I | No |
| I-control 8 | F | 25 | + | - | 0 | No |
| I-control 9 | F | 60 | - | - | 0 | No |
| I-control 10 | M | 41 | + | - | I | No |
| I-control 11 | F | 78 | - | - | 0 | No |
| I-control 12 | M | 44 | - | - | N.D. | No |
| I-control 13 | F | 32 | - | - | 0 | No |
| I-control 14 | M | 20 | - | - | 0 | No |
| I-control 15 | M | 16 | + | - | 0 | No |

Table shows the following information: gender (M: male and F: female), age (from 15 to 78 years), HLA DQ2/DQ8, IgA anti tTG (Anti-tissue transglutaminase antibodies), Marsh scale of the severity of lesion at diagnosis (from Marsh 0 to Marsh III) and patients on a Gluten-Free Diet (GFD). N.D. (Not determined).

**Table S4.** Clinical data from non-celiac disease patients without other inflamed conditions (C-controls, *n* = 25) from the Spanish population.

| **Sample** | **Gender** | **Age** | **HLA DQ2/DQ8** | **IgA**  **Anti-tTG** | **Marsh Criteria at Diagnosis** | **GFD** |
| --- | --- | --- | --- | --- | --- | --- |
| C-control 1 | F | 11 | - | - | 0 | No |
| C-control 2 | F | 28 | - | - | 0 | No |
| C-control 3 | F | 8 | - | - | 0 | No |
| C-control 4 | M | 76 | - | - | 0 | No |
| C-control 5 | F | 38 | - | - | 0 | No |
| C-control 6 | F | 21 | - | - | 0 | No |
| C-control 7 | F | 45 | - | - | 0 | No |
| C-control 8 | F | 62 | - | - | 0 | No |
| C-control 9 | M | 6 | - | - | 0 | No |
| C-control 10 | M | 70 | - | - | 0 | No |
| C-control 11 | M | 28 | - | - | 0 | No |
| C-control 12 | F | 17 | - | - | 0 | No |
| C-control 13 | F | 39 | - | - | 0 | No |
| C-control 14 | F | 34 | - | - | 0 | No |
| C-control 15 | M | 16 | - | - | 0 | No |
| C-control 16 | F | 81 | - | - | 0 | No |
| C-control 17 | F | 67 | + | - | 0 | No |
| C-control 18 | F | 15 | - | - | 0 | Yes |
| C-control 19 | M | 62 | - | - | 0 | No |
| C-control 20 | M | 13 | - | - | 0 | No |
| C-control 21 | F | 20 | - | - | 0 | No |
| C-control 22 | F | 31 | + | - | 0 | No |
| C-control 23 | M | 49 | - | - | 0 | No |
| C-control 24 | F | 44 | - | - | 0 | No |
| C-control 25 | F | 77 | - | - | 0 | No |

Table shows the following information: gender (M: male and F: female), age (from 6 to 81 years), HLA DQ2/DQ8, IgA anti tTG (Anti-tissue transglutaminase antibodies), Marsh scale of the severity of lesion at diagnosis (from Marsh 0 to Marsh III) and patients on a Gluten-Free Diet (GFD).

**Table S5.** Clinical data from untreated Celiac Disease patients (uCD, *n* = 20) from the Argentinian population.

| **Sample** | **Gender** | **Age** | **HLA DQ2/DQ8** | **IgA**  **Anti-tTG** | **Marsh Criteria at Diagnosis** |
| --- | --- | --- | --- | --- | --- |
| uCD 1 | F | 6 | + | + | N.D. |
| uCD 2 | F | 27 | + | + | III |
| uCD 3 | M | 11 | + | - (EMA+) | III |
| uCD 4 | F | 28 | + | + | II |
| uCD 5 | F | 39 | + | + | III |
| uCD 6 | F | 4 | + | + | III |
| uCD 7 | F | 10 | + | + | III |
| uCD 8 | M | 17 | + | + | N.D. |
| uCD 9 | F | 30 | + | + | III |
| uCD 10 | F | 15 | + | + | III |
| uCD 11 | F | 48 | + | - (EMA+) | III |
| uCD 12 | M | 25 | + | + | III |

**Table S5.** *Cont.*

| **Sample** | **Gender** | **Age** | **HLA DQ2/DQ8** | **IgA**  **Anti-tTG** | **Marsh Criteria at Diagnosis** |
| --- | --- | --- | --- | --- | --- |
| uCD 13 | M | 30 | + | + | II |
| uCD 14 | M | 42 | + | + | III |
| uCD 15 | F | 37 | + | + | III |
| uCD 16 | M | 32 | + | + | III |
| uCD 17 | F | 8 | + | + | III |
| uCD 18 | F | 56 | + | + | III |
| uCD 19 | F | 23 | + | + | II |
| uCD 20 | F | 9 | + | + | III |

Table shows the following information: gender (M: male and F: female), age (from 4 to 56 years), HLA DQ2/DQ8, IgA anti tTG (Anti-tissue transglutaminase antibodies) and Marsh scale of the severity of lesion at diagnosis (from Marsh I to Marsh III). N.D. (Not determined). EMA (Endomysium antibodies).

**Table S6.** Clinical data from non-celiac disease patients without other inflamed conditions (C-controls, *n* = 19) from the Argentinian population.

| **Sample** | **Gender** | **Age** | **HLA DQ2/DQ8** | **IgA**  **Anti-tTG** | **Marsh Criteria at Diagnosis** | **GFD** |
| --- | --- | --- | --- | --- | --- | --- |
| C-control 1 | F | 9 | - | - | 0 | No |
| C-control 2 | M | 15 | - | - | 0 | No |
| C-control 3 | F | 58 | - | - | 0 | No |
| C-control 4 | M | 24 | - | - | 0 | No |
| C-control 5 | F | 6 | - | - | 0 | No |
| C-control 6 | M | 37 | - | - | 0 | No |
| C-control 7 | M | 31 | - | - | 0 | No |
| C-control 8 | M | 62 | - | - | 0 | No |
| C-control 9 | M | 12 | - | - | 0 | No |
| C-control 10 | F | 33 | - | - | 0 | No |
| C-control 11 | M | 47 | - | - | 0 | No |
| C-control 12 | F | 50 | - | - | 0 | No |
| C-control 13 | F | 28 | - | - | 0 | No |
| C-control 14 | M | 62 | - | - | 0 | No |
| C-control 15 | F | 29 | - | - | 0 | No |
| C-control 16 | M | 36 | - | - | 0 | No |
| C-control 17 | M | 32 | - | - | 0 | No |
| C-control 18 | F | 17 | - | - | 0 | No |
| C-control 19 | F | 10 | - | - | 0 | No |

Table shows the following information: gender (M: male and F: female), age (from 6 to 62 years), HLA DQ2/DQ8, IgA anti tTG (Anti-tissue transglutaminase antibodies), Marsh scale of the severity of lesion at diagnosis (from Marsh 0 to Marsh III) and patients on a Gluten-Free Diet (GFD).

© 2015 by the authors; licensee MDPI, Basel, Switzerland. This article is an open access article distributed under the terms and conditions of the Creative Commons by Attribution (CC-BY) license (http://creativecommons.org/licenses/by/4.0/).
